# Prolonged oxidative stress and delayed tissue repair exacerbate acetaminophen-induced liver injury in aged mice

**DOI:** 10.18632/aging.103973

**Published:** 2020-10-01

**Authors:** Naoki Tanimizu, Norihisa Ichinohe, Hiromu Suzuki, Toshihiro Mitaka

**Affiliations:** 1 Department of Tissue Development and Regeneration, Research Institute for Frontier Medicine, Sapporo Medical University School of Medicine, Chuo-ku 060-8556, Japan; 2 Department of Molecular Biology, Sapporo Medical University School of Medicine, Chuo-ku 060-8556, Japan

**Keywords:** acetaminophen, drug-induced liver injury, aging, oxidative stress, necrosis

## Abstract

The liver gradually loses its regenerative capabilities with aging. However, it remains unknown whether aging affects drug-induced liver injury. Here, we used acetaminophen induced acute liver injury model to compare tissue injury and regeneration of aged mice (>80 weeks old) with young ones (8–10 weeks old). The mortality of aged mice after acetaminophen injury was higher than that of young mice. Transient increase of serum GOT and decrease of reduced glutathione (GSH) were not returned to original levels in aged mice even at 48 hours. In addition, *Foxm1b* and its targets *Ccnd1* and *Cdk1* were upregulated in young but not in aged mice after 48 hours. Moreover, an apoptosis-related gene, *Cidea,* was upregulated specifically in aged livers, which was consistent with increased number of TUNEL^+^ hepatocytes. Unexpectedly, damaged hepatocytes were retained in aged liver tissue, which may be caused by impaired recruitment of macrophages to the damaged area, without increases in *Ccl2* after acetaminophen injury. Collectively, prolonged oxidative stress due to delayed recovery of GSH and the retention of damaged hepatocytes may suppress tissue repair and hepatocyte proliferation, resulting in exacerbation of acetaminophen injury in aged mice. Thus, aging is a risk factor conferring susceptibility against drug-induced liver injury.

## INTRODUCTION

Upon tissue loss by acute liver injury or surgical resection, the liver shows strong regenerative capacity, in which the remaining hepatocytes grow and proliferate to restore the organ to its original size [1–3]. However, aging limits their regenerative capacity [4, 5]. An age-dependent decline in hepatocyte proliferation has been observed in a primary culture of hepatocytes [6] and in regeneration after partial hepatectomy (PHx) [7–11]. Several molecular mechanisms have been correlated with impaired proliferative capabilities in the aged liver. *Foxm1b* that regulates cell cycle-related genes [12, 13]; the DNA-binding capacity of Farnesoid X receptor (FXR), which is a factor upstream of *Foxm1b* [14] and YAP activity [15] are suppressed in the aged liver. The reduction of YAP activity downregulates budding uninhibited by benzimidazole-related 1 (*Bubr1*), which positively and negatively regulates the anaphase-promoting complex, and cellular senescence-related genes, including *p16^INK4a^*, respectively [16, 17]. In addition, extrinsic factors have been implicated in the age-dependent decrease of regenerative capacity. Reduction of growth hormone causes downregulation of GSK3β [18, 19], upregulation of p66^Shc^ inhibits the Ras/MAPK pathway [20, 21], and increase of interferon γ inhibits cell cycle progression through activation of p21 and nuclear accumulation of p53 [22–24]. However, it is not clear whether the regeneration capacity of aged livers is suppressed simply by the reduced proliferative capability of hepatocytes.

The liver is the central organ involved in metabolism, and most drugs are modified in hepatocytes, depending on activities of cytochrome P450 (CYPs). Drug derivatives could be toxic to hepatic cells and induce liver injury, which is called drug-induced liver injury (DILI). Acetaminophen (APAP) has analgesic and antipyretic properties and is widely used as a pain reliever and a fever reducer, though overdose of APAP causes fulminant hepatitis and, eventually, fatal liver failure [25].

The population of older individuals is continuously increasing worldwide. Recently, APAP medication has been preferable over nonsteroidal anti-inflammatory drugs (NSAIDs) as an analgesic when patients are suffering from gastrointestinal ulcers and kidney dysfunction [26]. Therefore, it is likely that older people will have more instances of APAP intake. Several studies suggested that aging itself is not a direct risk factor for the side effects of APAP, whereas others have pointed out that aging affects the rate of APAP clearance [27]. Characterization of APAP injury in aged mice is necessary for accumulation of experimental evidence to establish a safe use of APAP for the older population.

Here, we demonstrate that aged mice are more susceptible to APAP-induced fulminant liver damage. The mortality within 48 hours after APAP administration was increased in aged mice as compared with that in young mice. In addition to an impaired proliferative response in hepatocytes in aged mice, oxidative stress was prolonged by impaired activation of the reduced glutathione (GSH) synthetic pathway. Furthermore, hepatocytes underwent apoptosis instead of necrosis and the recruitment of macrophages to the damaged areas was impaired in aged mice, which might have led to delayed liver tissue repair and regeneration after APAP injury.

## RESULTS

### Prolonged liver tissue injury in aged mice after APAP administration

**Figure 1:**
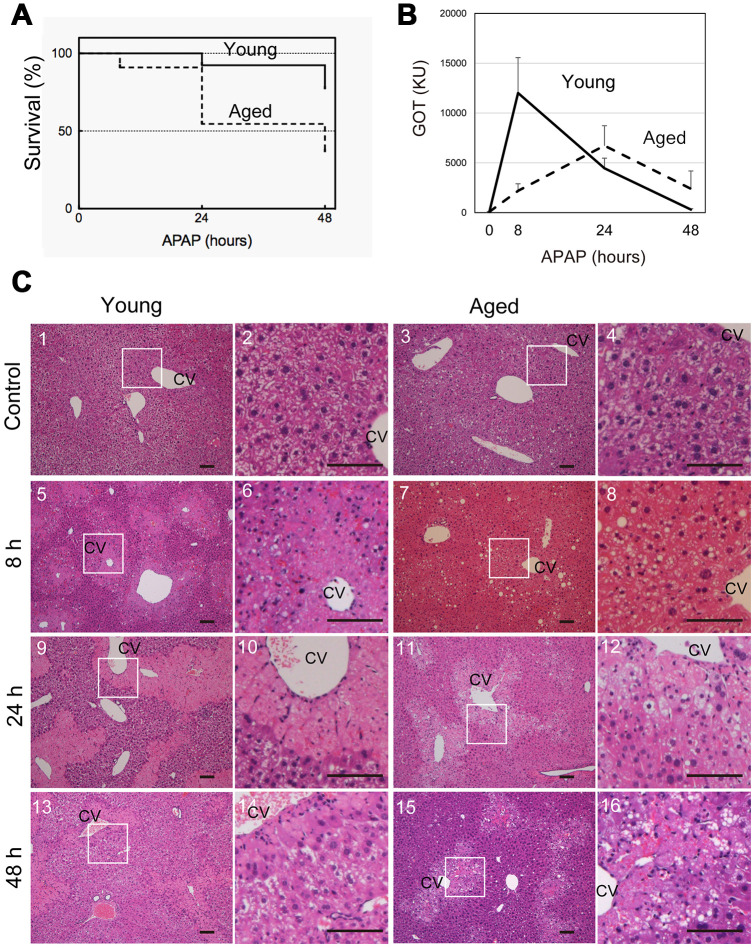
**Aged mice are more susceptible to acetaminophen-induced liver injury.** (**A**) Increased mortality by APAP injury in aged mice. Young (8-10W) and aged (>80W) mice were intraperitoneally injected with 300 mg/kg acetaminophen. N = 8 and 6 for young and aged mice, respectively. (**B**) Tissue damage is sustained at 48 hours after APAP injury in aged mice. Serum GOT increases at 8 hours after APAP administration, gradually decreases, and then gets back to the normal levels at 48 hours in young mice (solid line). By contrast, GOT is low at 8 hours and increases by 24 hours in aged mice (dotted line). GOT is still high at 48 hours. Serum was collected from more than six mice at each time point, and average values with SEMs are presented. (**C**) Histological analysis of young and aged liver tissue during APAP injury. APAP induces hepatocyte necrosis around CV, where hepatocytes lose their nuclei, and their cytoplasm is pale red with eosin staining (panels 5 and 6, 9 and 10). The damaged region is smaller at 48 hours after injury (panels 13 and 14). Contrastingly, in aged liver tissue, damaged areas are not clear at 8 hours (panels 7 and 8), become noticeable with pale eosin staining at 24 hours (panels 11 and 12), and persist at 48 hours (panels 15 and 16). Boxes in panels 1, 3, 5, 7, 9, 11, 13, and 15 are enlarged in panels 2, 4, 6, 8, 10, 12, 14, and 16, respectively. Bars represent 100 μm.

To examine whether the severity of APAP injury is affected by aging, young (8-10 weeks old) and aged (>80 weeks old) mice were intraperitoneally injected with 300 mg/kg APAP after starvation overnight. Forty-eight hours later, approximately 80% of young and 40% of aged mice survived at this APAP dose (Figure 1A). These data suggest that aged mice are more susceptible to APAP than young ones. To evaluate the effects of APAP on hepatocytes, we measured serum glutamate-oxaloacetate transaminase (GOT) levels over time, after APAP administration (Figure 1B). Serum GOT was remarkably increased after 8 hours and then gradually decreased to reach normal levels within 48 hours in young mice (solid line in Figure 1B). By contrast, GOT levels in aged mice gradually increased and peaked at 24 hours, after which the levels decreased but remained higher at 48 hours than those in controls without injury (dotted line in Figure 1B). We also examined glutamate-pyruvate transaminase (GPT) levels. Similar to GOT, they were already high at 8 hours in young but not in aged mice (Supplementary Figure 1). Based on the time course of GOT, we hypothesized that APAP-induced liver injury may be sustained in aged mice.

**Figure 2:**
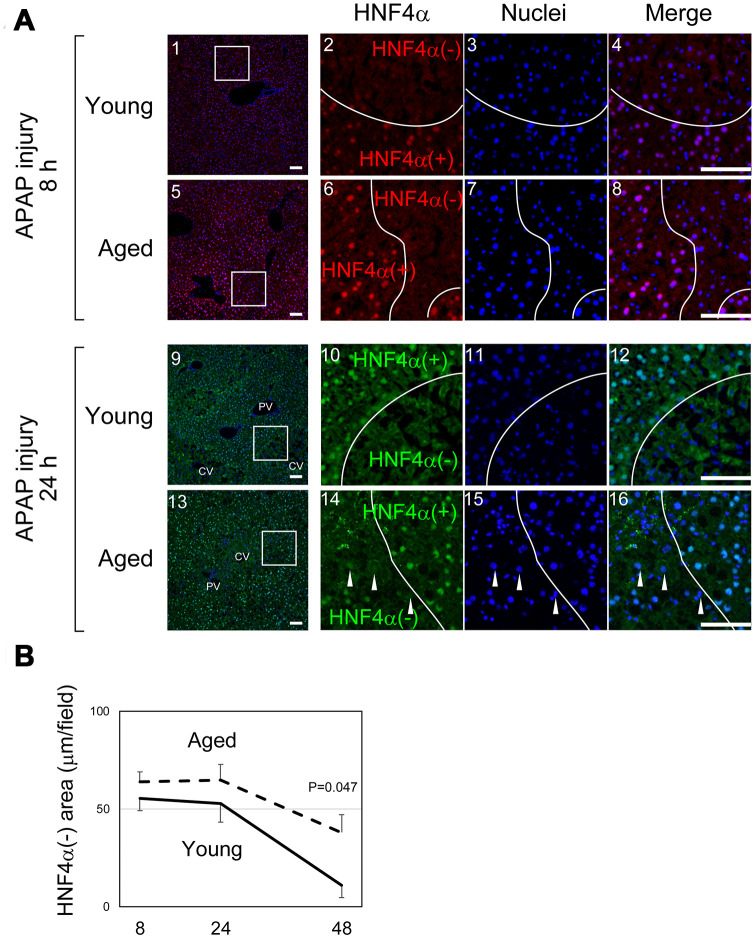
**Liver tissue damage is sustained in aged mice.** (**A**) Hepatocytes around CVs lose HNF4α. HNF4α is thoroughly and exclusively expressed in hepatocytes in the adult liver. Both in young and aged livers, hepatocytes around CVs that are damaged by APAP lose nuclear HNF4α, whereas hepatocytes outside the damaged tissue thoroughly express HNF4α. Bars represent 100 μm. (**B**) Quantification of the HNF4α region. The damaged areas containing HNF4α^-^ hepatocytes occupy approximately 50% of liver tissue at 8 and 24 hours after APAP administration in both young and aged mice. However, HNFα^-^ areas are greatly reduced in young but not in aged mice at 48 hours. Average values with SEMs are presented in the graph.

**Figure 3:**
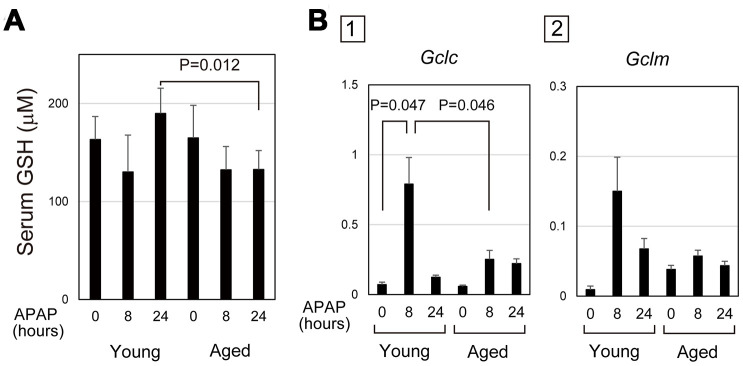
**Defective glutathione synthesis in aged livers after APAP injury.** (**A**) GSH oxidative stress is sustained in aged mice. Serum GSH is reduced at 8 hours after APAP administration in both young and aged mice. GSH levels are back to normal levels at 24 hours in young but not in aged mice. The graph shows average values with SEMs. (**B**) Expression of glutamate-cysteine ligase (GCL). *Gclc*, GCL catalytic subunit, is significantly upregulated at 8 and 24 hours after APAP administration in young mice. *Gclm*, the GCL modifier subunit, tends to be upregulated at 24 hours. By contrast, both *Gclc* and *Gclm* are not upregulated in aged mice. The graph shows average values with SEMs.

**Figure 4:**
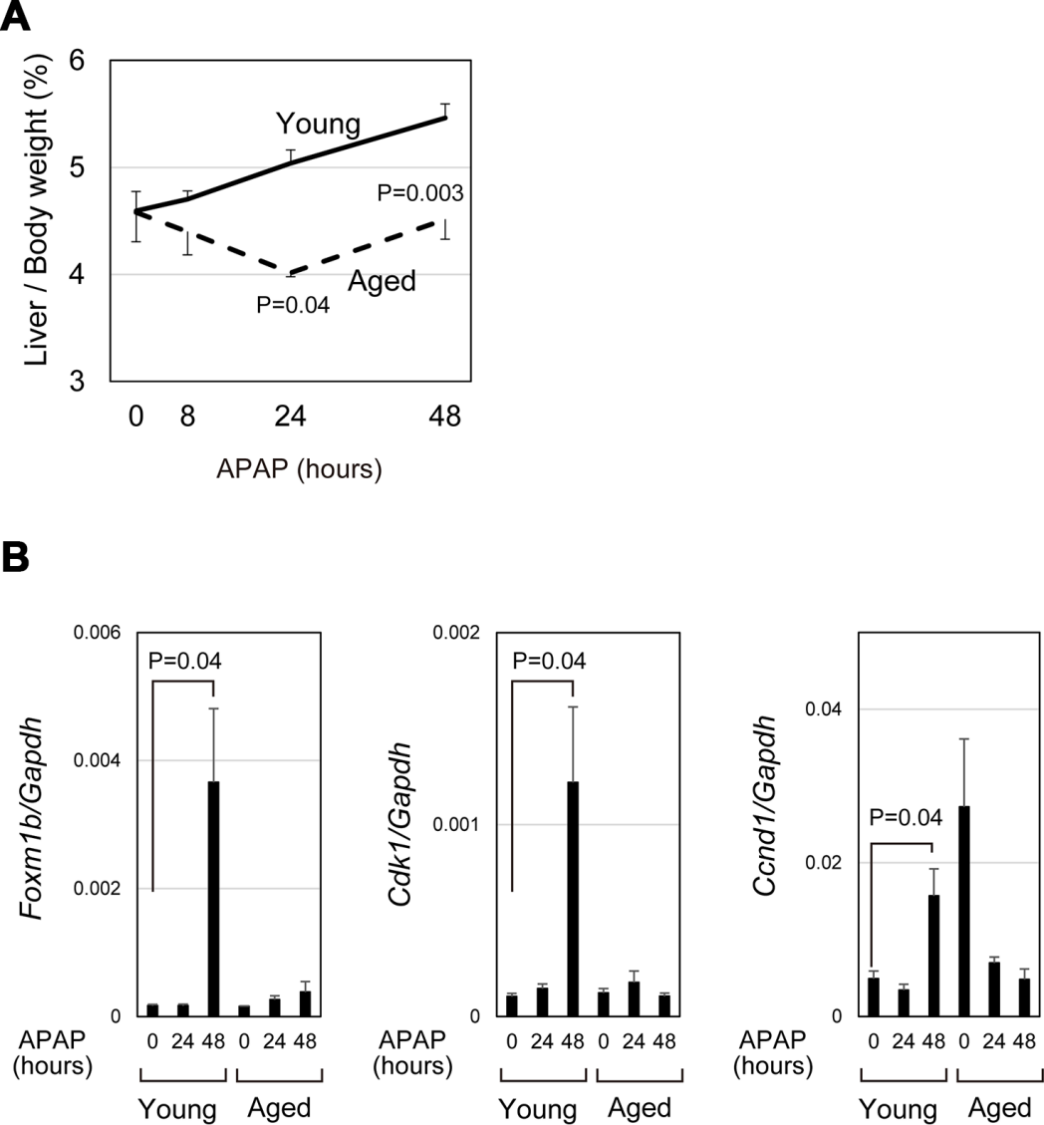
**qPCR analysis demonstrated impaired induction of cell-cycle-related genes.** (**A**) Impaired gain of liver weight in aged mice after APAP injury. Liver/body weight gradually increased in young mice after APAP administration, whereas it is once decreased at 24 hours after the injury and then recovered to the original ratio at 48 hours in aged mice. The difference of liver/body weight between young and aged mice is statistically significant at 24 and 48 hours. The graph shows average values with SEMs. (**B**) Expression analysis for genes involved in cell cycle progression. *Foxm1b*, *Cdk1*, and *Ccnd1* are upregulated at 48 hours after APAP injury in young livers but not in aged ones. The graph shows average values with SEMs.

**Figure 5:**
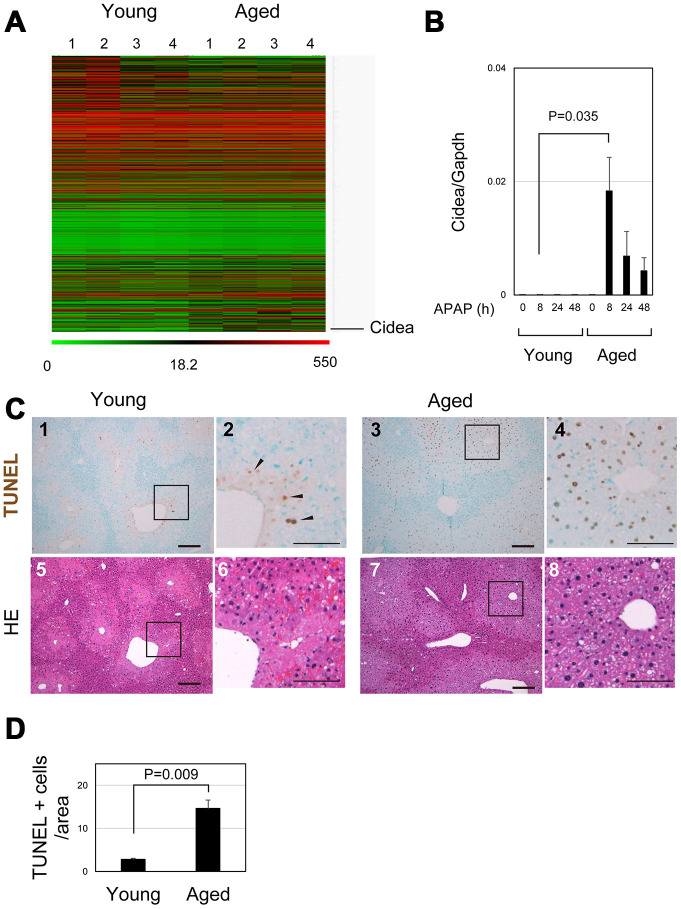
**Apoptosis is promoted in aged liver.** (**A**) Global gene expression profiles of young and aged livers at 8 hours after APAP injury. *Cidea* is listed as the most upregulated gene in aged livers 8 hours after APAP administration. Gene expression profiles were analyzed using microarray analysis. (**B**) *Cidea* is induced in aged liver tissue after APAP injury. Quantitative PCR analysis demonstrates that *Cidea* is significantly upregulated at 8 hours after APAP injury, specifically in aged livers. The graph shows average values with SEMs. (**C**) TUNEL staining of young and aged livers at 8 hours after APAP injury. APAP induces hepatocyte necrosis around the CV, and dead hepatocytes are mostly de-nucleated in young mice at 8 hours. In addition, a small number of TUNEL^+^ hepatocytes exist in the necrotic area. By contrast, hepatocytes around CV still possess their nuclei (panels 7 and 8), and they are mostly TUNEL^+^ (panels 3 and 4) in aged mice. Bars in panels 1, 3, 5, and 7, and panels 2, 4, 6, and 8 represent 50 and 100 μm, respectively. (**D**) Increase of TUNEL^+^ hepatocytes in aged livers. TUNEL^+^ hepatocytes within the distance of 100 μm from the CV is more in aged livers than those in young ones.

**Figure 6:**
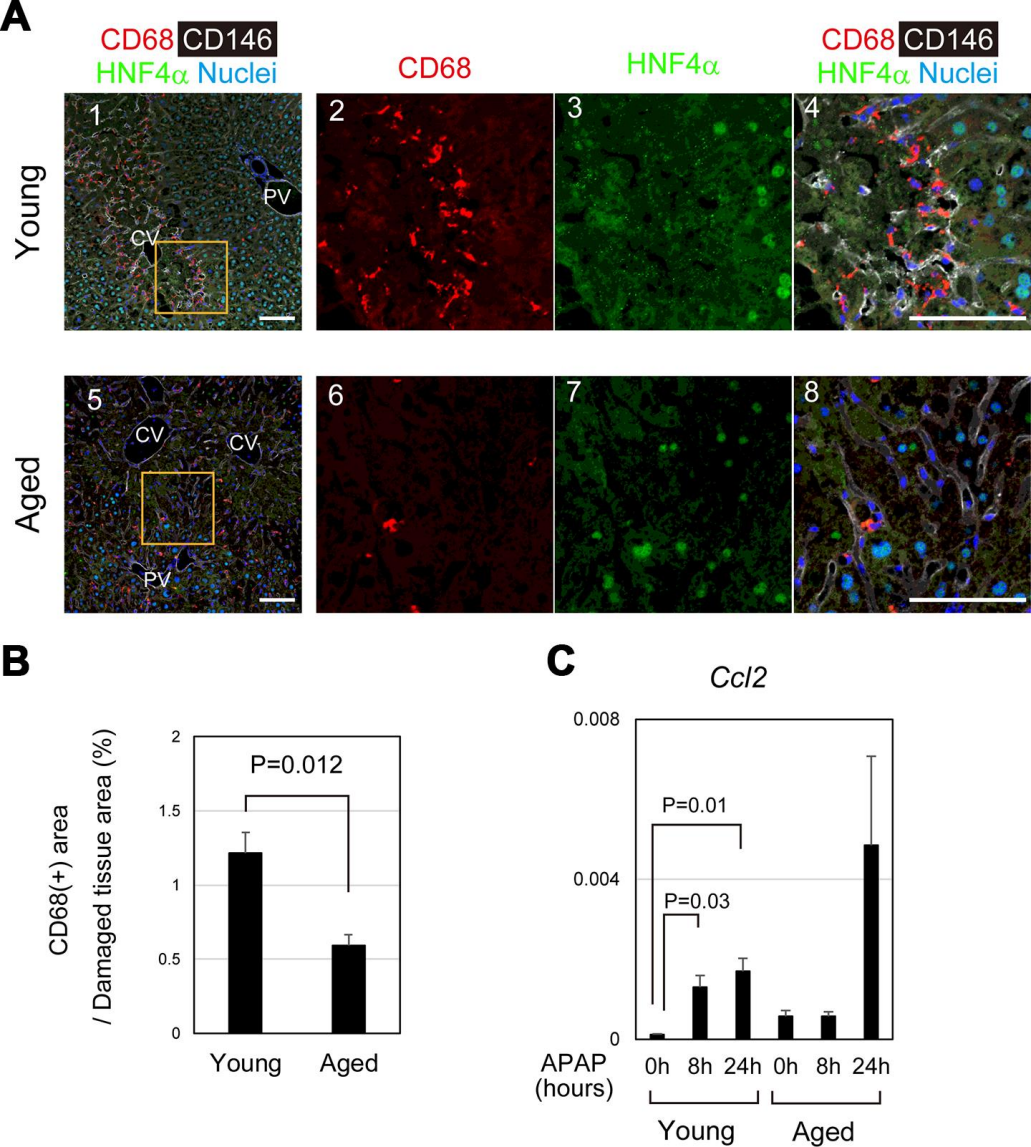
**Impaired recruitment of macrophages in aged mice after APAP injury.** (**A**) Macrophages accumulate toward the damaged area in young mice after APAP injury. CD68^+^ macrophages are abundant in damaged liver tissue around the CV at 24 hours in young mice (panels 1–4). Contrastingly, CD68^+^ macrophages in the damaged area of aged liver are minimal (panels 5–8). Scale bars in panels represent 100 μm. (**B**) Impaired recruitment of macrophages to damaged liver tissue in aged mice. CD68^+^ macrophages in the damaged tissue are significantly less in aged livers compared to those in young ones. Liver tissue consisting of HNF4α^-^ hepatocytes was damaged by APAP administration. The recruitment of macrophages was estimated from the ratio of the CD68^+^ area in the HNF4α^-^ tissue at 24 hours after APAP administration. Two areas were quantified on Image J. The graph shows average values with SEMs. (**C**) Induction of *Ccl2* expression. *Ccl2*, chemokine-attracting macrophages, is upregulated in young mice at 8 and 24 hours after APAP administration with statistical significance. Conversely, it is not significantly induced in aged mice. The graph shows average values with SEMs.

**Figure 7:**
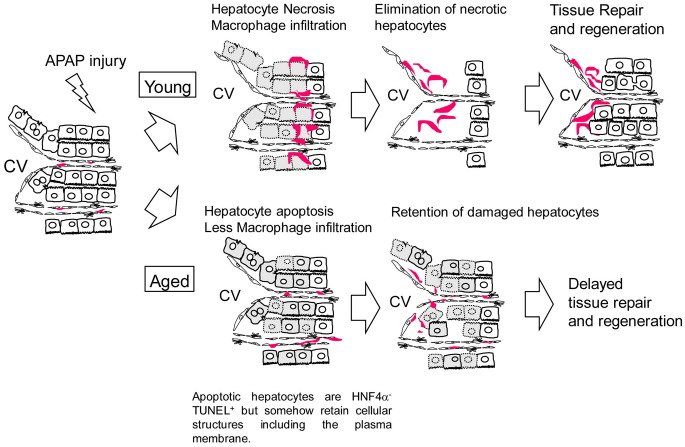
**Model for the responses against APAP injury in young and aged mice.** In young mice, APAP induces hepatocyte necrosis around the CV. Necrotic hepatocytes are eliminated by infiltrated macrophages, which is followed by hepatocyte proliferation. In aged mice, APAP induces hepatocyte apoptosis. Apoptotic hepatocytes are HNF4α^-^TUNEL^+^ but somehow retain cellular structures including the plasma membrane even at 24 h after the injury, suggesting they are slowly dying. In addition, macrophages are not efficiently recruited to the damaged tissue containing apoptotic hepatocytes. Consequently, the clearance of nonfunctional hepatocytes is delayed and the subsequent hepatocyte proliferation is suppressed in aged mice.

To further clarify the differences between young and aged liver tissue after APAP injury, liver tissues were histologically assessed using H&E staining. Eight hours after APAP administration in young mice, hepatocytes around the central vein (CV) underwent necrosis and could be distinguished from healthy hepatocytes as de-nucleated cells (Figure 1C-5 and 6). The area containing necrotic cells shows pale eosin staining (Figure 1C­5 and 6) and was more clearly recognized by the sharp contrast to the surrounding uninjured areas containing healthy hepatocytes, whose cytoplasm had dark eosin staining at 24 hours (Figure 1C-9 and 10). Although the necrotic areas remained at 48 hours, the areas became remarkably narrow (Figure 1C-13 and 14). In aged mice, necrotic areas were not clearly recognized through H&E staining at 8 hours (Figure 1C-7 and 8), and damaged hepatocytes became distinguishable from surrounding hepatocytes at 24 hours, based on paler cytosolic staining (Figure 1C-11 and 12). Of note, most of damaged hepatocytes still contained nuclei. The damaged areas were still prominent at 48 hours after APAP administration (Figure 1C-15 and 16).

As illustrated in Figure 1C, it was difficult to quantify the areas of damaged hepatocytes in aged liver only using H&E staining, in particular at 8 hours after APAP administration. To compare the size of the damaged tissue in aged livers with the size of damaged tissue in young livers, we used immunostaining with an anti-HNF4α antibody (Figure 2A). HNF4α is exclusively expressed in hepatocytes in an adult liver, and its strong expression indicates that hepatocytes are healthy and functional. Therefore, HNF4α(-) areas contain hepatocytes with reduced function due to APAP injury. HNF4α(-) areas were clearly identified both in young and aged livers at 8 hours and 24 hours (Figure 2A). As seen in the H&E staining, HNF4α(-) hepatocyte nuclei were detected in aged livers, even at 24 hours (arrowheads in Figure 2A-14–16). A quantitative analysis demonstrated that HNF4α(-) areas were comparable in size between young and aged livers at 24 hours, but that they were larger in aged livers at 48 hours (Figure 2B). We also performed immunostaining for glutamine synthetase (GS), which is normally expressed only around CVs. We expected GS might disappear after APAP administration and then be recovered along tissue repair. However, GS expression retained at least by 24 hours after APAP injury both in young and aged livers (Supplementary Figure 2-1–16). This result suggests that, although HNF4α(-) hepatocytes around the CVs are not functional, they still retain some cellular contents including GS. Collectively, the liver tissue damage induced by APAP administration was not initially greater in the aged mice, but the damage was sustained for a longer period of time in the aged mice.

### Defective glutathione synthesis in aged livers after APAP injury

Absorbed APAP is mostly glucuronidized or sulphonated in hepatocytes, but the remnant is converted into N-acetyl-p-benzoquinone imine (NAPQI) by Cyp2E1, which is conjugated with GSH for detoxification [28]. After APAP administration, the consumption of GSH in hepatocytes results in a transient decrease in GSH, which is recovered by recycling and synthesis. Therefore, the level of serum GSH may reflect the degree and duration of oxidative stress in the cells. In fact, the content of GSH in liver tissue after APAP injury was similar to the time course of serum GSH (Figure 3A and Supplementary Figure 3). In young mice, serum GSH transiently decreased at 8 hours after APAP administration and then reverted to the normal levels at 24 hours. However, serum GSH at 24 hours stayed at a similar level as at 8 hours after APAP administration in aged mice. We considered a possibility that delayed GSH recovery could be caused by impaired GSH synthesis.

Glutamate-cysteine ligase (GCL) is the rate-limiting enzyme for GSH synthesis. A quantitative PCR analysis demonstrated that GCL catalytic subunit (*Gclc*) was significantly upregulated 8 hours after APAP administration in young, but not in aged, livers (Figure 3B-1). Additionally, expression of GCL modifier subunit (*Gclm*) was relatively higher in young livers at 8 hours compared with the control (Figure 3B-2). However, *Gclm* upregulation was highly variable in each mouse. Therefore, the defective activation of the GSH synthetic pathway caused by impaired induction of *Gclc* may result in the delayed recovery of GSH in aged mice. This may lead to prolonged oxidative stress, which may sustain APAP-induced injury in aged mice.

### Decreased proliferative response in APAP-injured aged liver

APAP caused severe acute liver damage but liver/body weight was gradually increased in young mice by 48 hours (bold line in Figure 4A). However, in aged mice, liver/body weight was reduced at 24 hours after APAP treatment and only slightly increased between 24 and 48 hours (dotted line in Figure 4A). We compared the expression of cell-cycle-related genes in aged livers with the expression in young livers. Consistent with the previous reports that hepatocyte proliferation is apparent at 48 hours after APAP injury [29, 30], *Foxm1b* and its downstream targets, *Cdk1* and *Ccnd1*, were upregulated at 48 hours in young mice (Figure 4B). In contrast, those cell cycle related genes were not upregulated in aged mice, though two out of five aged mice had high expression of *Ccnd1* without injury. In consistent with PCR data, hepatocytes strongly positive for PCNA were evident near the CV at 48 hours in young, but not in aged, livers (closed arrowheads in Supplementary Figure 2-17–20). These data demonstrate that hepatocyte proliferation is not induced in the aged liver at least 48 hours after APAP injury. Thus, even if mice survive under the prolonged oxidative stress, liver regeneration does not proceed normally in aged mice.

### Damaged hepatocytes undergo apoptosis in aged liver

To identify crucial events in aged livers after APAP injury, the gene expression profiles of young and aged livers at 8 hours after APAP injury were examined by microarray analysis (Figure 5A and Supplementary Table 2). Cell-death-inducing DNA fragmentation factor (DFF) 45-like effector A (*Cidea)* was listed as the most upregulated gene in aged liver. Quantitative PCR analysis further confirmed that *Cidea* was significantly upregulated 8 hours after APAP administration, specifically in aged mice (Figure 5B). *Cidea* has been correlated with induction of apoptotic cell death [31]. Although necrosis is the major cell death pathway after APAP treatment, apoptosis is also observed in the APAP-injured liver [32–34]. Although some TUNEL^+^ cells were observed around the CV of young liver at 8 hours (arrowheads in Figure 5C-1 and 2), the number of TUNEL^+^ hepatocytes around the CV was much greater in aged liver tissue than that in young one (Figure 5C-3 and 4 and 5D). Thus, damaged hepatocytes mostly undergo necrosis and apoptosis in young and aged livers, respectively. Apoptotic cell death of pericentral hepatocytes in aged mice may explain why their nuclei were retained at least by 24 hours after APAP insult (Figure 1C-11 and 12, 2B-5–8, 5C-7 and 8). This is in sharp contrast to necrotic hepatocytes in young livers (Figure 5C-5 and 6).

Surprisingly, even at 24 hours, the plasma membrane of hepatocytes around the CV was partly disturbed but still maintained in aged liver (arrowheads in Supplementary Figure 4). This suggests that apoptosis in the aged liver may proceed very slowly. The last step of apoptotic cell death may be disturbed or delayed, resulting in prolonged tissue injury and delayed tissue regeneration.

### Sustained liver injury in aged mice

As demonstrated in Figure 1B, GOT was still high at 48 hours after APAP injury, and GSH was not back to normal levels at 24 hours in aged mice (Figure 3A). In addition, damaged hepatocytes were retained in the injured liver tissue of aged mice at 24 hours (Figures 1C, 2, and 5C). Furthermore, the pericentral zone was still positive for GS in aged mice even at 48 hours (Supplementary Figure 2-15 and 16), suggesting that damaged hepatocytes remained. This is contrast to the liver tissue of young mice, which was partly devoid of GS staining at 48 hours (Supplementary Figure 2-13 and 14). Moreover, hepatocyte proliferation was not induced at 48 hours in aged liver (Figure 4 and Supplementary Figure 2-19-20). These results indicate that tissue repair does not properly proceed, and, therefore, tissue damage is protracted in aged liver after APAP injury, which may block hepatocyte proliferation.

Dead hepatocytes are phagocytosed by macrophages and cleared from the area, which may be prerequisite for the remaining hepatocytes proliferate and restore the lost tissue. In young mice, CD68^+^ macrophages accumulated toward damaged hepatocytes, particularly those in the boundary between necrotic and healthy areas at 24 hours after APAP administration (Figure 6A-1–4). By contrast, macrophages were apparently fewer in pericentral area of aged livers than that of young ones (Figure 6A-5–8). Quantitative analysis further demonstrated that the recruitment of macrophages to the damaged tissue was significantly suppressed in the aged liver (Figure 6B). Consistently, *Ccl2*, a macrophage-attracting chemokine, was upregulated at 8 and 24 hours after APAP administration in young but not in aged liver tissue (Figure 6C). Without statistical significance, *Ccl2* was increased at 24 hours in aged mice, because *Ccl2* was high in one aged mouse. Although CD68^+^ macrophages were abundant in its liver tissue, they were not accumulated in the damaged area (Supplementary Figure 5). Collectively, the recruitment of macrophages to the damaged liver tissue is impaired in aged mice, which results in retention of damaged hepatocytes and therefore may suppress tissue repair and regeneration.

## DISCUSSION

APAP is an effective analgesic, but its overdose causes severe liver injury. However, it remains unknown how aging affects APAP-induced liver injury. Here, we implicated sustained oxidative stress and impaired recruitment of macrophages into the damaged tissue in prolonged liver tissue damage and delayed liver regeneration after APAP injury in aged mice.

Reduction of regenerative capacity of the aged liver is partly elucidated by decreased hepatocyte proliferative capability [5]. Consistently, cell-cycle-related genes were not induced in aged livers after APAP injury (Figure 4). Absence of hepatocytes strongly positive for PCNA at 48 hours further indicates that hepatocyte proliferation was not induced in aged livers (Supplementary Figure 2-17–20). In addition to the possibility that aged hepatocytes have reduced proliferative capabilities, our data suggest that the way of hepatocyte death and extrinsic factors are involved in impaired liver regeneration. APAP is known to induce hepatocyte necrosis. By contrast, we found that many hepatocytes underwent apoptosis instead of necrosis in the aged liver. Many of the apoptotic hepatocytes maintained their plasma membrane at least 24 hours after APAP injury. In addition, macrophage infiltration into the damaged area was not promoted, likely because of impaired induction of *Ccl2*. Thus, we consider that slow apoptotic cell death and defective recruitment of macrophages delay tissue repair and subsequent hepatocyte proliferation in the aged liver after APAP injury.

We hypothesized that there may be two potential reasons for impaired macrophage infiltration in aged livers after APAP injury. One is that there is no induction of *Ccl2*, and the other is that the slowly dying hepatocytes may not be efficiently recognized by the innate immune system. Additionally, reduced accumulation of macrophages in damaged tissue could be directly correlated with impaired tissue repair and regeneration in aged mice after APAP injury. Monocyte-derived macrophages, but not resident Kupffer cells, promptly accumulate in damaged tissue after APAP injury [35]. Blocking the infiltration of these macrophages with administration of a CCR2 inhibitor reduced liver tissue damage, indicating that monocyte-derived macrophages promote the acute phase of APAP-induced injury. By contrast, those macrophages eventually convert their pro-inflammatory characteristics into pro-resolving ones, and inhibition of this conversion resulted in prolonged tissue damage and delayed regeneration [36]. Therefore, it is possible that less macrophage accumulation in aged mice results in delayed tissue repair and regeneration.

In addition to hepatocyte proliferation, many factors should be considered to understand liver injury and regeneration of aged livers. The older population may also suffer from obesity and have livers with senescent hepatocytes, low levels of inflammation, and continuous exposure to reactive oxygen (ROS). These chronic conditions can increase the risks, exacerbating APAP-induced liver injury. In particular, hepatic steatosis is correlated with the level of APAP-induced liver damage [37]. Steatosis increases CYP2E1-mediated production of NAPQI, which causes mitochondrial deficits of GSH and enhanced activation of JNK. However, we did not find a drastic reduction in GSH in aged mice compared with young ones without APAP injury (Figure 3A and Supplementary Figure 3). A recent study demonstrated that fat accumulation in aged hepatocytes increases macrophage infiltration and leads to low levels of tissue injury, which is indicated by increases in serum injury markers [38]. Although we noticed that aged mice gained weight associated with expanded adipose tissue, their serum GOT values were quite normal before APAP administration (Supplementary Figure 6), indicating that despite obvious weight gain and increased fat tissue, aged mice maintain liver functions and do not have any apparent chronic conditions, at least in our experiments.

Cellular senescence has been correlated with aging [39]. Senescent hepatocytes gradually accumulate in aged livers and continuously release senescent-associated secretory proteins (SASP), which increase inflammatory cytokines and suppress regenerative responses [40]. Therefore, if senescent hepatocytes are abundant in the aged normal liver, they certainly affect responses against APAP. We performed senescence-associated beta-galactosidase (SA-βGal) staining and did not find many senescent hepatocytes in normal young and aged livers (Supplementary Figure 7). SA-βGal^+^ hepatocytes were slightly increased after APAP injury around the CV of aged livers. It could be possible that those senescent hepatocytes secrete SASP and sustain tissue injury. However, given that hepatocytes outside the CV area that are not affected by APAP are involved in regeneration, we consider that hepatocyte senescence is not likely to play a major role in suppressing regenerative response in the aged liver after APAP injury.

Accumulation of ROS is correlated with age–related disease development [41, 42]. We assessed oxidative status in normal and APAP-injured aged livers by measuring GSH (Figure 3 and Supplementary Figure 3). Our results indicate that GSH levels were similar in normal young and aged livers, but the recovery from reduced GSH due to APAP insult was impaired in aged mice. Since *Gclc* is not rapidly induced in aged mice after injury, the primary reason for lower recovery of GSH may be impaired induction of GSH synthesis-related gene expression. Therefore, the capability eliminating ROS from liver tissue is insufficient to combat continuous liver damage in aged mice.

In this work, we demonstrate that APAP-induced liver injury is prolonged in aged mice. Sustained oxidative stress and suppressed hepatocyte proliferation may cause higher mortality after APAP administration in aged mice. Additionally, the retention of dead hepatocytes may block the initiation of tissue regeneration. The mechanism of delayed regeneration due to APAP administration in aged mice is illustrated in Figure 7. Collectively, this work demonstrates a potential risk for giving APAP to aged patients.

## MATERIALS AND METHODS

### Mice

C57/BL6 mice were purchased from Sankyo Labo Service Corporation, Inc. (Tokyo, Japan). Acetaminophen (Sigma-Aldrich, St. Louis, MO) dissolved in warm PBS at 10 mg/ml was administrated to mice through intraperitoneal injection (300 mg/kg). Blood was collected from the tail vein or inferior vena cava and the serum was isolated by centrifugation. Activity of GOT was measured using the Transaminase CII Test Wako (Wako Pure Chem., Osaka, Japan). All animal experiments were approved by the Sapporo Medical University Institutional Animal Care and Use Committee and were conducted according to institutional guidelines for ethical animal use.

### Quantification of glutathione

Glutathione concentration was measured by ELISA using a glutathione detection kit (ENZO Life sciences, Inc., Farmingdale, NY) according to the manufacturer’s instructions.

### Histochemical analysis and TUNEL staining

Normal and injured liver tissues fixed in PBS containing 4% paraformaldehyde were embedded in paraffin. Paraffin sections were used for hematoxylin-eosin (H&E) or TdT-mediated UTP nick-end labeling positive (TUNEL) staining. TUNEL staining was performed using an *in situ* apoptosis detection kit (Takara Bio Inc., Kusatsu, Japan) according to the manufacturer’s protocol. Images were collected on an Olympus IX70 microscope (Olympus Co. Ltd., Tokyo, Japan).

### Immunostaining and confocal imaging

Normal and injured liver tissues were fixed in PBS containing 4% paraformaldehyde at 4 °C. After embedding in OCT compound, samples are frozen in -80°C until use. Seven μm-thick frozen sections were prepared on a cryostat (Leica, Wetzlar, Germany). After washing with PBS, samples were permeabilized in PBS containing 0.2% Triton X-100 at room temperature for 5 minutes. After blocking in Blockace (DS pharma biomedicals Co. Ltd., Osaka, Japan), they were incubated with rabbit anti-HNF4α (1:600 dilution, Santa Cruz Biotechnologies, Dallas, TX), rat anti-mouse CD68 (1:500 dilution, Biolegend, San Diego, CA), and rabbit anti-mouse CD146 (1:1,000 dilution, a gift from Dr. Yamato Kikkawa, Tokyo University of Pharmacy and Life Sciences, Japan). After being washed in PBS, sections were incubated with AlexaFluor-dye-conjugated secondary antibodies (Thermo Fisher Scientific, Waltham, MA). Nuclei were counterstained with Hoechst33342 (Dojindo Laboratories, Kumamoto, Japan). Images were acquired using a Zeiss LSM780 or Nikon A1 confocal laser scanning microscope. The damaged areas containing HNF4α^-^ hepatocytes and the macrophage invasion area were quantified on Olympus cellSens software and on Image J, respectively. Unpaired two-tailed *t*-tests were performed using Microsoft Excel.

For staining for glutamine synthetase (GS) and proliferating cell nuclear antigen (PCNA), paraffin sections were prepared. After de-paraffinization in xylene, sections were treated in Target Retrieval Solution (Dako, Jena, Germany). Sections were incubated with mouse anti-human GS (1:200 dilution, BD biosciences, San Jose, CA) or mouse anti-PCNA (1:200 dilution, Dako) antibodies at 4°C overnight. Signals were detected using VECTASTAIN ABC kit (Vector Laboratories Inc., Burlingame, CA).

### Quantitative PCR

Total RNA extracted from frozen tissue was used for cDNA synthesis. Quantitative PCR was performed on ABI7500. The primers used are listed in Supplementary Table 1. Sample numbers were N = 3, 8, 4, 4 at 0, 8, 24, and 48 hours, respectively, for young mice, whereas N = 3, 4, 4, 4 for aged mice. Unpaired two-tailed *t*-tests were performed using Microsoft Excel.

### Microarray analysis

Liver tissue samples were collected from 8-week (n = 4) and 80-week old mice (n = 4) at 8 hours after APAP administration. Total RNA extracted from frozen liver tissues was used for labeling and then hybridized onto the SurePrint G3 Mouse Gene Expression Microarray (Agilent Technologies, Santa Clara, CA). Data was deposit in GEO (https://www.ncbi.nlm.nih.gov/geo/query/acc.cgi?acc=GSE149740).

## Supplementary Material

Supplementary Figures

Supplementary Tables
