# Supplementary material for: Prolonged oxidative stress and delayed tissue repair exacerbate acetaminophen-induced liver injury in aged mice
Source: Aging (Albany NY). 2020 Oct 1;12(19):18907–27. doi: 10.18632/aging.103973 (PMC7732315; doi:10.18632/aging.103973)
Supplement: Supplementary Figures [file aging-12-103973-s001..pdf]

SUPPLEMENTARY FIGURES

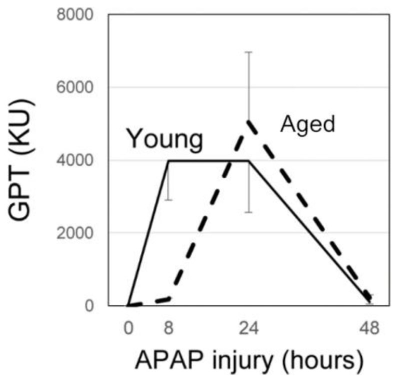

Supplementary Figure 1. Change of GPT during APAP injury. Serum GPT increases at 8 and 24 hours after APAP administration in young mice (solid line). By contrast, GPT is low at 8 hours and increases by 24 hours in aged mice (dotted line). Serum was collected from more than six mice at each time point, and average values with SEMs are presented.

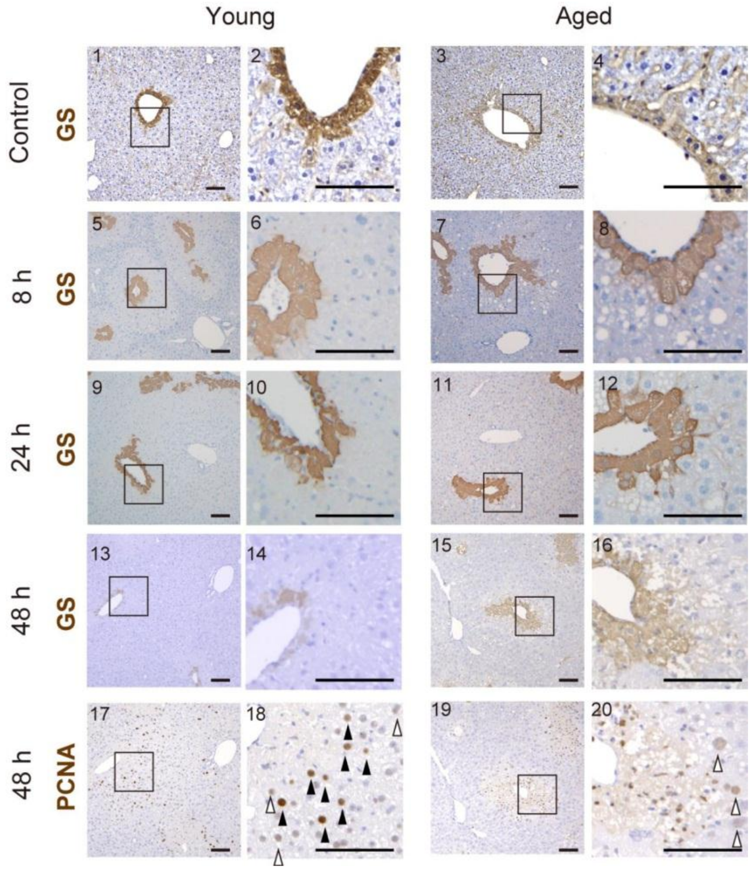

Supplementary Figure 2. Expression of GS and PCNA after APAP injury. GS expression in the pericentral zone retained after APAP injury by 24 hours after APAP administration. At 48 hours, it partly disappears in young mice, suggesting that damaged hepatocytes in this area are eliminated by this time point. At 48 hours, hepatocytes strongly positive for PCNA are evident in young but not in aged mice (closed arrowheads in panel 18). In addition, hepatocytes weakly positive for PNCA are observed both in young and aged mice (open arrowheads in panels 18 and 20). Bars represent 100 µm.

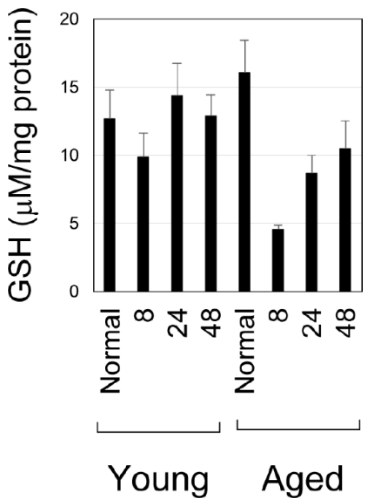

Supplementary Figure 3. Change of cellular GSH during APAP injury. GSH in liver tissue transiently decreases in young livers and is rapidly back to the normal level by 24 h. On the other hand, reduced GSH only slowly increases between 8 and 48 h but not returns to the normal level even at 48 h in aged mice. Cell extract was obtained by homogenizing frozen liver tissue in the presence of protease inhibitor cocktail. GSH and protein concentration were measured using GSH assay kit and BCA assay kit, respectively.

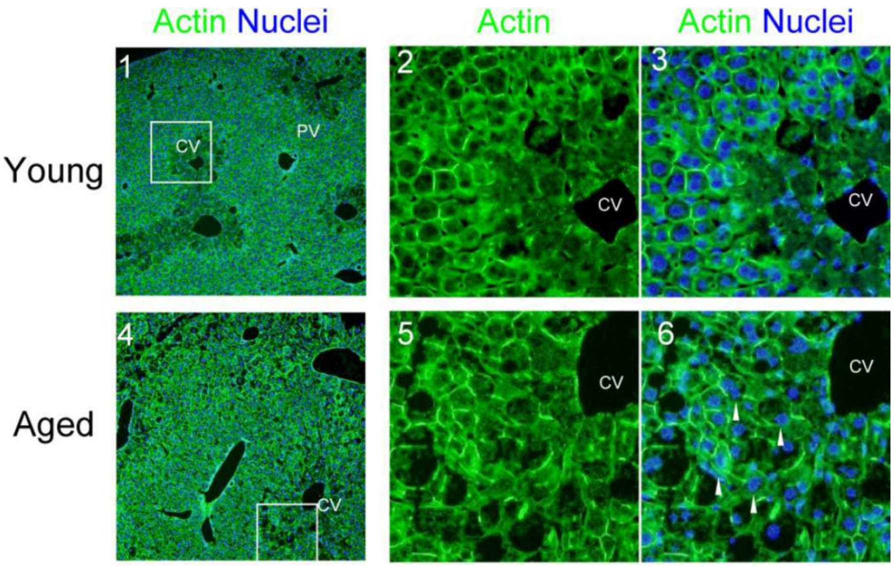

Supplementary Figure 4. Pericentral damaged hepatocytes retain the plasma membrane in aged liver at 24 h after APAP administration. AlexaFluor 488-phalloidin staining shows that most hepatocytes in pericentral areas lose actin bundles beneath cell membrane and those hepatocytes are without nuclei (panels 1–3). On the other hand, aged hepatocytes near the CV retain nuclei and the plasma membrane (white arrowheads in panel 6).

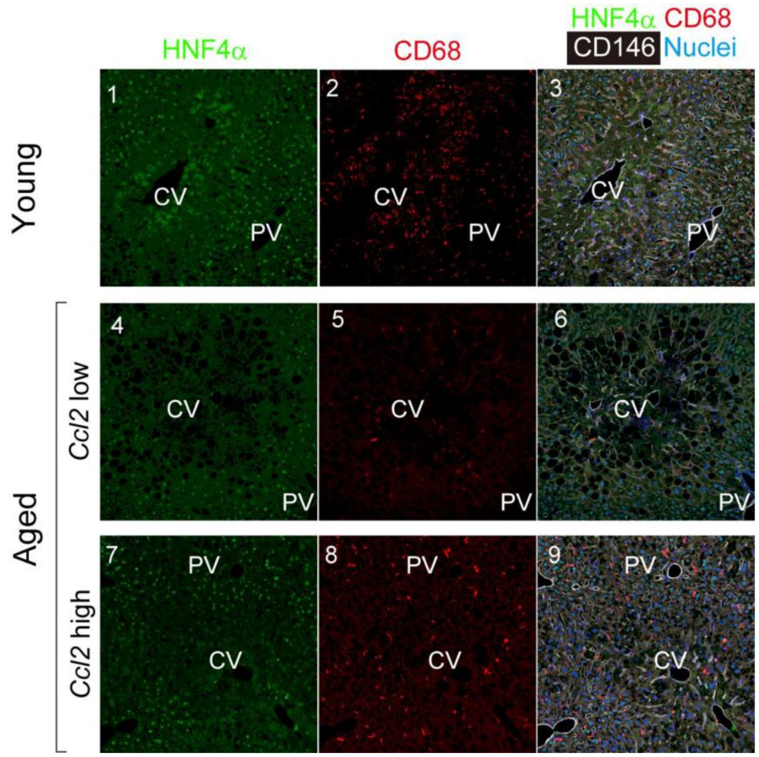

Supplementary Figure 5. Localization of CD68$^+$ macrophages in young and *Ccl2* high aged mice. At 24 hours after APAP injury, CD68$^+$ macrophages are accumulated in the damaged tissue around the CV in a young mouse (panels 1–3). CD68$^+$ macrophages are less in the *Ccl2* low aged mouse (panels 4–6). On the other hand, CD68$^+$ macrophages are abundant in the *Ccl2* high aged mouse, but they are not accumulated in the damaged tissue around the CV (panels 7–9).

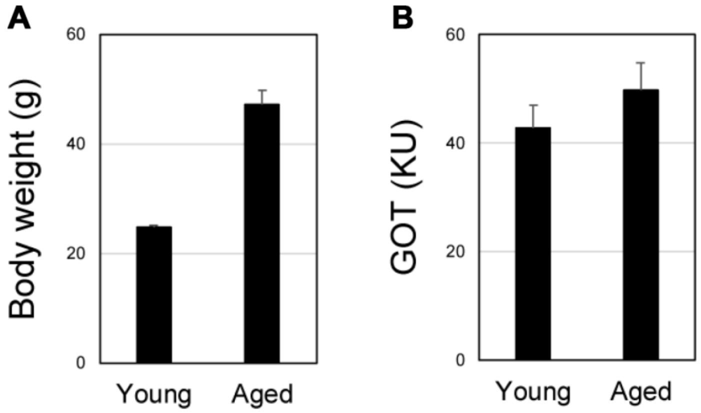

Supplementary Figure 6. Aged mice gain weight. (**A**) Aged mice are heavier than young ones. (**B**) Aged mice do not show increased serum GOT without injury.

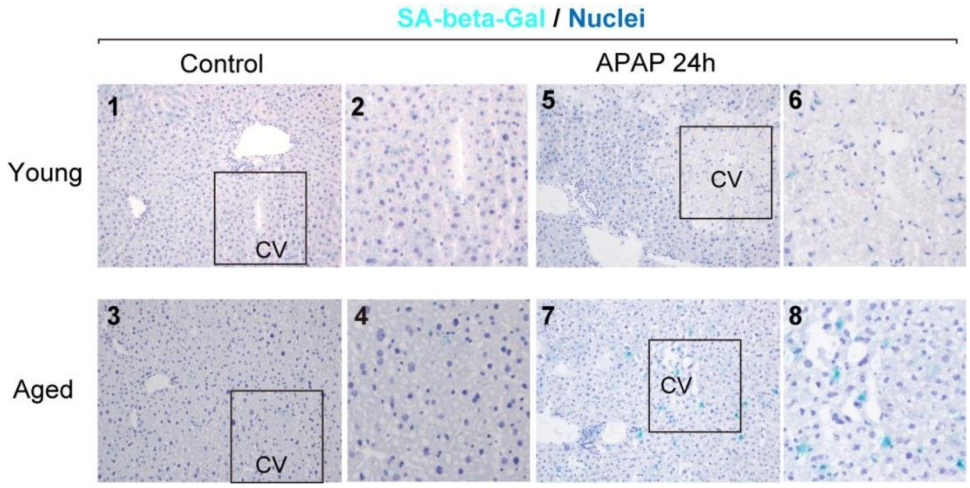

Supplementary Figure 7. Detection of senescence associated β-galactosidase (SA-βGal) positive cells in young and aged livers. SA-βGal$^+$ hepatocytes are observed in liver tissue neither of young and aged mice (panels 1–4). A few SA-βGal$^+$ cells are detected in young liver after injury. On the other hand, SA-βgal$^+$ hepatocytes are evident in aged liver but their location is mostly limited in the area around the CV (panels 7 and 8). Boxes in panels 1, 3, 5, and 7 are enlarged in panels, 2, 4, 6, and 8, respectively.
