# Supplementary material for: Prolonged oxidative stress and delayed tissue repair exacerbate acetaminophen-induced liver injury in aged mice
Source: Aging (Albany NY). 2020 Oct 1;12(19):18907–27. doi: 10.18632/aging.103973 (PMC7732315; doi:10.18632/aging.103973)
Supplement: Supplementary Tables [file aging-12-103973-s002..pdf]

SUPPLEMENTARY TABLES

Supplementary Table 1. PCR primers.

Gene Name		Sequence
Ccl2	Forward	catccacgtgttggctca
	Reverse	gatcatcttgctggtgaatgagt
Ccnd1	Forward	tttctttccagagtcatcaagtgt
	Reverse	tgactccagaagggcttcaa
Cdk1	Forward	gaacttcgacatccaaatatagtcag
	Reverse	ccatggacaggaactcaaaga
Cidea	Forward	ttcaaggccgtgttaagga
	Reverse	cctttggtgctaggcttgg
Foxm1b	Forward	gtgtgcctgttcccaagc
	Reverse	ctgttgtccagcgtgcag
Gapdh	Forward	ttgcagtggcaaagtggaga
	Reverse	gatgggcttcccgttgatga
Gclc	Forward	agatgatagaacacgggaggag
	Reverse	tgatcctaaagcgattgttcttc
Gclm	Forward	tgactcacaatgacccgaaa
	Reverse	tcaatgtcagggatgctttct

Supplementary Table 2. Apoptosis related genes.

	80W vs 8W	p value
Cidea	5.90E+02	0.0210
Dnase1	1.00E+01	0.0001
Fasl	3.07E+00	0.0002
Apaf1	2.11E+00	0.3420
Cidec	2.01E+00	0.1075
Casp7	1.83E+00	0.0394
Gadd45a	1.80E+00	0.1226
Bag4	1.73E+00	0.0407
Cradd	1.67E+00	0.0475
Fas	1.67E+00	0.1224
Bcl2l11	1.64E+00	0.4575
Hrk	1.62E+00	0.0941
Bnip3	1.36E+00	0.2315
Casp2	1.32E+00	0.4504
Tnfsf8	1.27E+00	0.3642
Abl1	1.24E+00	0.4461
Cideb	1.24E+00	0.2029
Bnip1	1.15E+00	0.4798
Cd40lg	1.14E+00	0.0012
Cd70	1.13E+00	0.0083
Dapk1	1.13E+00	0.4762
Bcl2l10	1.12E+00	0.0108
Bik	1.12E+00	0.6250
Bfar	1.04E+00	0.7785
Dffa	1.04E+00	0.8963

Cell cycle related genes

	80W vs 8W	p value
Cdkn2a	1.01E+01	0.0056
Cnnm1	1.84E+00	0.0670
Cdkn1a	1.59E+00	0.0803
Foxm1	1.51E+00	0.2065
Cdk5	1.09E+00	0.5315
Cdkn2d	1.08E+00	0.5314
Ccnd3	9.20E-01	0.2873
Cdkn2c	6.74E-01	0.3577
Cdk1	6.41E-01	0.1331
Pcna	6.41E-01	0.1440
Ccne1	6.10E-01	0.1636
Cdkn2b	6.04E-01	0.2163
Mki67	5.84E-01	0.1640

Pericentral genes

	80W vs 8W	p value
Lgr5	9.60E+00	0.0769
Glul	3.89E+00	0.1020
Gys2	2.23E+00	0.0053
Cldn2	2.08E+00	0.1799
Cdh2	1.64E+00	0.0710
Cyp7a1	1.63E+00	0.4824

Cyp1a2	1.57E+00	0.2326
Axin2	1.14E+00	0.0112
Cyp2e1	9.97E-01	0.9737
Ctnnb1	5.81E-01	0.2772

Cytokines

	**80W vs 8W**	**p value**
Ifng	2.74E+00	0.0020
Il6	1.81E+00	0.3888
Il10	1.43E+00	0.3465
Il4	1.12E+00	0.0098
Il13	7.40E-01	0.2462
Il1b	5.86E-01	0.4956
Tnf	5.78E-01	0.0070

Macrophages related genes

	80W vs 8W	p value
Mmd2	1.41E+01	0.0150
Mmd	2.69E+00	0.0235
Csf2rb	2.47E+00	0.0609
Fbxo10	1.41E+00	0.3297
Mgl2	1.15E+00	0.2905
Mif	8.92E-01	0.5669
Maea	6.16E-01	0.3548
Ccl2	2.11E-01	0.1624

Hepatocyte markers

	**80W vs 8W**	**p value**
Alb	9.78E-01	0.6964
Hnf1a	1.15E+00	0.5963
Hnf4a	1.19E+00	0.4310
Onecut1	1.71E+00	0.3873
Onecut2	1.06E+00	0.8492
Cebpa	1.35E+00	0.3524
G6pc	2.55E+00	0.0308
Cps1	5.69E-01	0.0334
Fabp1	9.61E-01	0.7649
Pck1	1.78E+00	0.0510
Tat	1.25E+00	0.4722
Tdo2	1.13E+00	0.7164

Cholangiocyte markers

	**80W vs 8W**	**p value**
Prom1	1.24E+00	0.3024
Epcam	5.15E-01	0.4227
Tacstd2	8.22E-01	0.6844
Krt7	2.33E-01	0.3136
Krt19	1.52E-01	0.3160
Spp1	6.55E-01	0.5702
Hes1	9.26E-01	0.5371
Hnf1b	9.60E-01	0.8835
Sox9	6.38E-01	0.2353
